# Spine malformation complex in 3 diverse syndromic entities: Case reports

**DOI:** 10.1097/MD.0000000000005505

**Published:** 2016-12-16

**Authors:** Ali Al Kaissi, Andreas van Egmond-Fröhlich, Sergey Ryabykh, Polina Ochirov, Vladimir Kenis, Jochen G. Hofstaetter, Franz Grill, Rudolf Ganger, Susanne Gerit Kircher

**Affiliations:** a Ludwig Boltzmann Institute of Osteology, the Hanusch Hospital of WGKK and AUVA Trauma Centre Meidling, First Medical Department, Hanusch Hospital; b Orthopaedic Hospital of Speising, Paediatric Department; c Department of Pediatrics, Kaiser-Franz-Josef Spital, Vienna, Austria; d Axial Skeleton and Neurosurgery Department, Restorative Traumatology and Orthopaedics, Ilizarov Center, Kurgan, Russia; e Pediatric Orthopedic Institute n.a. H. Turner, Department of Foot and Ankle Surgery, Neuroorthopaedics and Systemic Disorders, Saint-Petersburg, Russia; f Institute of Medical Chemistry, Medical University of Vienna, Austria.

**Keywords:** Acampomelic campomelic dysplasia, case reports, Larsen syndrome, Morquio syndrome type A, phenotype/genotype, spinal malformation

## Abstract

**Rationale:**

Clinical and radiographic phenotypic characterizations were the base line tool of diagnosis in 3 syndromic disorders in which congenital cervico-thoracic kyphosis was the major deformity.

**Patients concerns:**

Directing maximal care toward the radiographic analysis is not only the axial malformation but also toward the appendicular abnormalities was our main concern. We fully documented the diversity of the spine phenotypic malformation complex via the clinical and radiographic phenotypes.

**Diagnoses:**

We established the diagnosis via phenotypic/genotypic confirmation in 3 diverse syndromic entities namely acampomelic campomelic dysplasia, Larsen syndrome and Morquio syndrome type A (mucopolysaccharidosis type IV A).

**Interventions:**

Surgical interventions have been carried out in the Larsen syndrome and Morquio syndrome type A, resepectively.

**Outcomes:**

The earliest the diagnosis is, the better the results are. The necessity to diagnose children in their first year of life has many folds, firstly the management would be in favor of the child's growth and development and secondly, the prognosis could be clearer to the family and the medical staff as well. Our current paper is to sensitize paediatricians, physicians and orthopedic surgeons regarding the necessity to detect the aetiological understanding in every child who manifests a constellation of malformation complex.

**Lesons:**

Scoliosis and kyphosis/kyphoscoliosis are not a diagnosis in themselves. Such deformities are mostly a symptom complex correlated to dozens of types of syndromic associations. The rate curve progression and the final severity of congenital spine tilting are related to 3 factors: (a) the type of vertebral malformation present, (b) the patient's phenotype, and (c) the diagnosis.

## 1 Introduction

Campomelic dysplasia (CD, Online Mendelian Inheritance in Man no. 114290) is a rare, often lethal, autosomal dominant osseous malformation syndrome. This type of skeletal dysplasia is characterized by bowing of the femur and tibia, short first metacarpals, hypoplastic scapulae, non-mineralization of the pedicles of the thoracic vertebrae, presence of 11 pairs of ribs, narrow iliac wings, and poor ossification of the pubis. Additional problems identified in long-term survivors include short stature, cervical spine instability with cord compression, progressive scoliosis, and hearing impairment.^[1]^ The Acampomelic campomelic dysplasia (ACD) is a rare clinical variant of the more commonly encountered CD, characterized by absence of long bone curvature (acampomelia).^[2,3]^ To date, it has been shown that both, CD and ACD, can be caused by heterozygous mutations in the *SOX9*-gene or chromosomal abberrations such as translocations, inversions or deletions affecting the *SOX9*-gene, or the putative enhancer region.^[4]^ The gene product SOX9-protein controls genes responsible for the normal development of different organ systems such as the skeleton and the reproductive system.

Larsen et al^[5]^ drew attention to a syndrome of multiple congenital dislocations associated with a characteristic facies. Larsen syndrome is a rare, pathologic condition, characterized by multiple joint dislocations, distinctive deformities of the hands and feet, characteristic facial features described as a “dish face,” with a saddle nose and hypertelorism, kyphoscoliosis, and segmentation anomalies of the vertebrae (Online Mendelian Inheritance in Man no. 150250). Diverse treatment options, including conservative observation and surgical correction, have been reported for patients who present with cervical spine pathophysiology.^[6]^ The responsible *FLNB*-gene has been identified on chromosome 3p14.3.^[7]^ The same gene is mutated in atelosteogenesis types I and III, and in spondylocarpotarsal syndromes. Mutations seem to cluster in about 5 of the 46 exons.^[8]^ The gene product filamin B plays a role in the cytoskeleton and network of protein filaments such as actin, which allows actin and other proteins to adapt the cytoskeleton to changes in shape and growth during development.

Mucopolysaccharidosis type IV A (MPS IVA or Morquio syndrome type A, Online Mendelian Inheritance in Man no. 253000) is also a rare autosomal recessive hereditary disorder caused by an enzyme deficiency of N-acetylgalactosamine-6-sulfatase (GALNS). This enzyme is encoded by the *GALNS*-gene on chromosome 16q24.3. Infants with MPS IV seem to be normal at birth, but by the age of 18 to 20 months, they develop characteristic clinical features affecting multiple organ systems, mainly containing connective tissue.^[9,10]^ GALNS is responsible for the degradation of keratan-sulfate and chrondroitin-6-sulfate, which are the major components of proteoglycans in the cartilage and the bone. Therefore, skeletal dysplasia is the major manifestation in MPS IV A in affected children, known as dysostosis multiplex.^[10]^ Aplasia or hypoplasia of varying degrees of the dens axis or the os odontoideum can be observed in nearly all patients, causing effectively the development of spinal cord compression due to anterior soft tissue mass and indentation by the posterior arch of the atlas. Many patients are at risk for developing cervical myelopathy and quadriplegia due to atlantoaxial subluxation from odontoid hypoplasia and ligamentous hyperlaxity.^[11,12]^

## 2 Clinical reports

### 2.1 Acampomelic campomelic dysplasia

**Figure 1:**
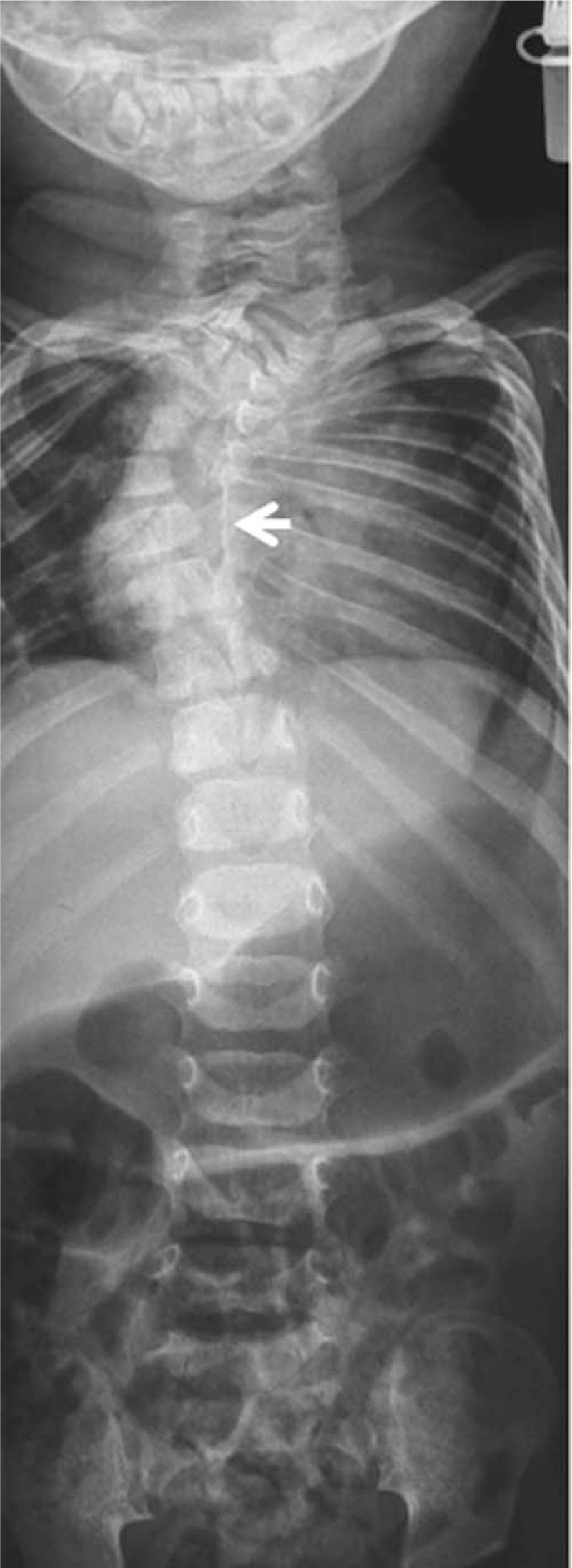
AP radiograph of the spine showed cervico-thoracic kyphoscoliosis with a Cobbs angle of 90°. Note the puzzle like cervico-thoracic spine associated with underdeveloped pedicles (arrow). AP radiograph = Anterior-posterior radiograph.

**Figure 2:**
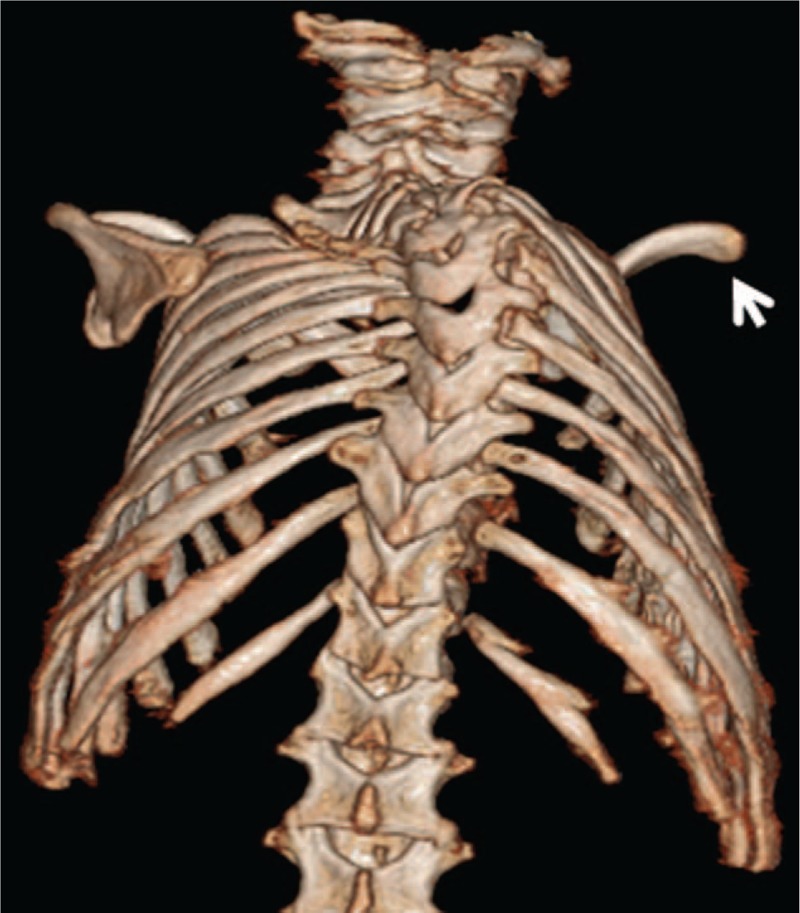
3D-CT scan in a 2-year-old girl with acampomelic campomelic dysplasia showed significant disconnection of the posterior spine elements, related to underdeveloped pedicles and hypoplasia of the laminae along different cervical levels C2/4 and C6, associated with extensive malsegmentation and associated with the complete absence of the right scapula (arrow). 3D-CT = three-dimensional computer tomography.

A-2-year old girl was presented the first time in our clinic: at birth, her clinical examination showed Pierre Robin sequence associated with congenital cervico-thoracic kyphosis and floppiness. The child had bouts of nasal regurgitation while swallowing, associated with nasal escape of air (heard as grunting) because of her cleft palate. Ultrasound examination of the brain, heart, and kidneys were normal. She underwent palatal pushback repair of her cleft palate, and bilateral myringotomy and tube placement were undertaken at the same time. In her early life, there were attempts to reduce her kyphosis by means of extension and a closed reduction. The outcome, however, was unpleasant. Examination at the age of 2 years showed cervicothoracic kyphosis with Cobb's angle of 90°. After thorough clinical examination to detect specific criteria of cutaneous midline lesions suggestive of spinal dysraphism such as a hairy tuft, dimple, or hemangioma were not present in our patient. Anterior-posterior (AP)-radiograph of the spine showed cervico-thoracic kyphoscoliosis with a Cobbs angle of 90° and the puzzle like cervico-thoracic spine associated with underdeveloped pedicles(arrow) (Fig. 1). Three-dimensional computer tomography (3D-CT)-scan reconstructions showed significant disconnection of the posterior spine elements related to underdeveloped pedicles and hypoplasia of the laminae along different cervical levels C2/4 and C6 associated with extensive mal-segmentation associated with the complete absence of the right scapula (arrow) (Fig. 2).

Neurological examination was consistent with moderate spinal cord injury at C7/T1. There was paresis of the trunk and legs associated with acceptable finger movement and full elbow and wrist flexion and extension. The head movement was satisfactory as well as the shoulder movement. At the age of 10 years, the patient had a cervical kyphotic deformity with a Cobb's angle of 135°. Noninstrumented anterior and posterior fusion with halo-cast immobilization was suggested. The medical team and the parents preferred not to perform spinal surgery because of the neurological risk. She required invasive ventilation almost 24 hours per day because of thoracic and therefore respiratory insufficiency syndrome. Her incomplete paraplegia has improved and she is able to walk short distances and attends school. The child showed a de novo mutation in the *SOX9*-gene (missense mutation in the DNA-binding domain (p.Pro170Arg)).

### 2.2 Larsen syndrome

**Figure 3:**
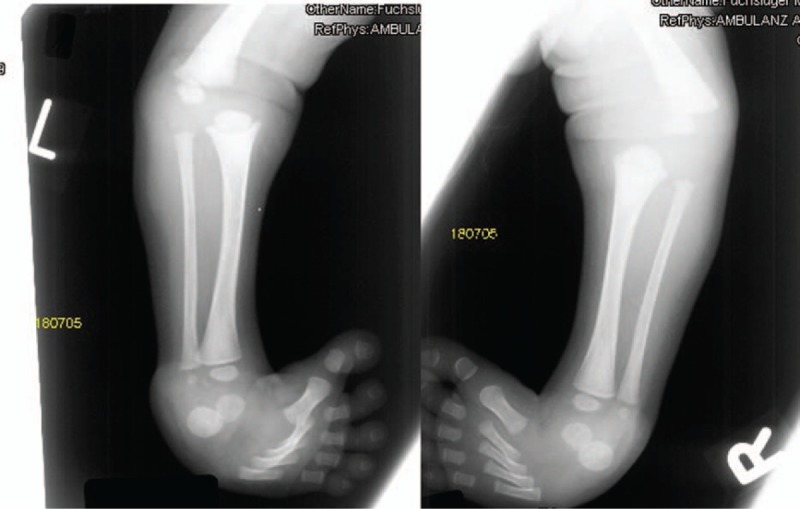
Radiograph of the lower limbs showed bilateral and symmetrical involvement of clubfoot, bilateral dislocation of the knees (most characteristically anterior dislocation of the tibia on the femur).

**Figure 4:**
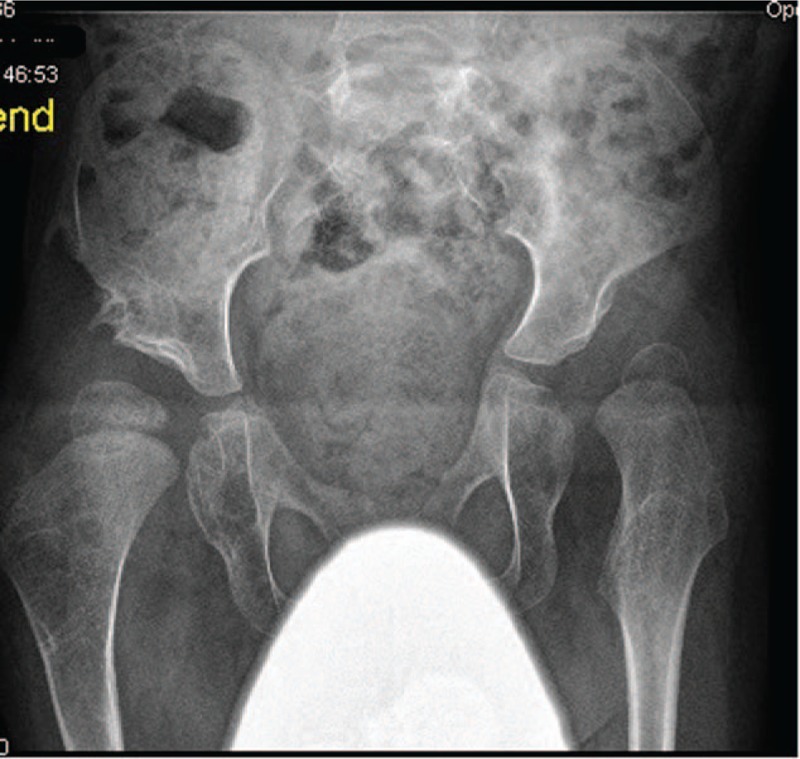
AP radiograph of the pelvis showed bilateral dislocation of the hips associated with massive acetabulo-femoral dysplasia. AP radiograph = Anterior-posterior radiograph.

A male child was introduced to our department: at birth his growth parameters were minus 2 standard deviations (SD). Parents were healthy and nonconsanguineous, the family history was unremarkable. At birth, he presented with a peculiar dysmorphic facial features (dish-like face) associated with cleft palate. Apparent cervicothoracic kyphosis associated with multiple contractures were the notable deformities. Skeletal survey showed bilateral and symmetrical involvement of clubfoot, bilateral dislocation of the knees with, most characteristically, anterior dislocation of the tibia on the femur (Fig. 3). Additional findings were the bilateral dislocation of the hips associated with massive acetabulo-femoral dysplasia (Fig. 4). Short metacarpals with cylindrical fingers lacking the usual tapering were the prime skeletal features, associated with under-mineralization and over-tubulation of the long bones. Extra-calcaneal ossification centre appeared in his late infancy. Supernumerary carpal bones associated with lack of distal tapering of the proximal and middle phalanges were noticeable radiographic features. Genetic tests showed mutation of the *FLNB*-gene (p.G1691S).

**Figure 5:**
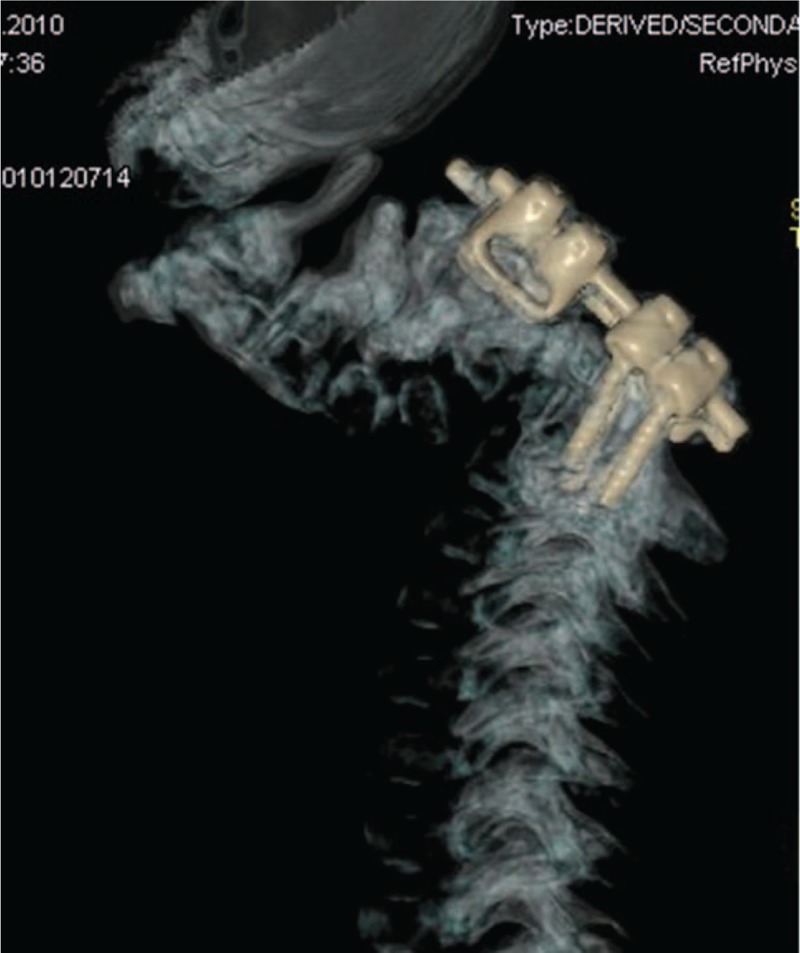
3DCT scan in a 2-year-old boy with the Larsen syndrome showed dorsal spinal fusion and laminectomy of C3–C7. 3D-CT = three-dimensional computer tomography.

Primarily, orthopaedic intervention to correct his multiple dislocations has been started at age of 2 months. The patient underwent overhead traction followed by arthrography and spica cast for 5 weeks. A hip flexion splint was applied. At age of 8 months, a percutaneous dorsal release of the Achilles tendon was performed. At age of 2 and a half year, an open reduction of the right hip with a femoral varisation and derotation as well as Pemberton osteotomy was performed and hardware was removed later. At that time, progression of the kyphosis to 90° had occurred. In addition, significant hypoplasia of the cervical vertebral bodies along the cervical spine segments from C5 to C7 has been recognized. In order to arrest the progressive kyphosis, the child underwent laminectomy of C5-C7 with dorsal spinal fusion (spondylodesis) of C3–7 (Fig. 5).

### 2.3 Morquio syndrome type A

**Figure 6:**
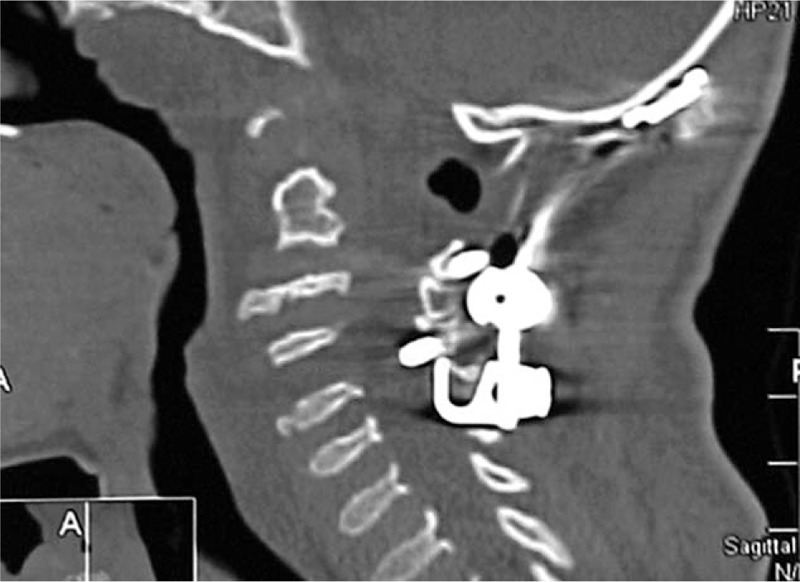
3DCT scan in a 7-year-old girl with MPS type IV A showed laminectomy of C1 was done at the II stage of surgical treatment (occipitospondylodesis). Correction and posterior instrumentation of the cervical spine at the C0–C5 level in conjunction with posterior spinal fusion with costal autograft was performed. Few months after decompression at C1 level, occipitospondylodesis (i.e., fixation of the upper 2 or 3 cervical vertebrae to the occipital bone) was performed. 3D-CT = three-dimensional computer tomography, MPS = mucopolysaccharidosis.

**Figure 7:**
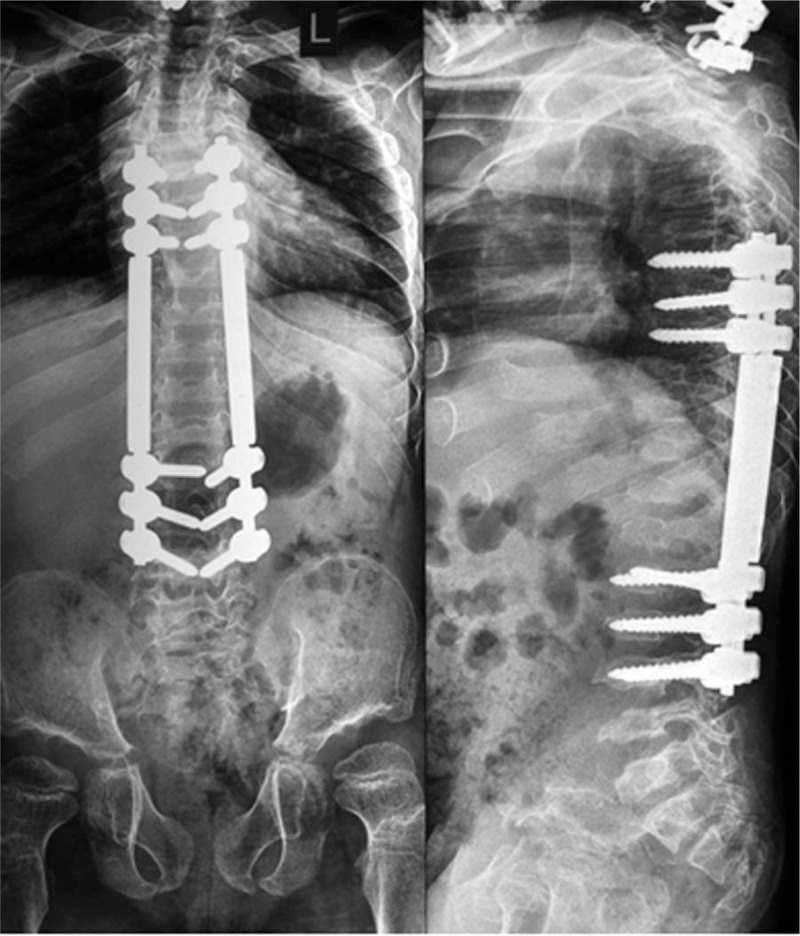
The above-mentioned procedures in the Morquio A-patient were followed by correction of the thoraco-lumbar kyphosis, done via posterior spinal fusion at the level of Thoracic spine 6-Lumbar spine 4 by means of “growing rods” system.

A 7 years-old girl was referred to our department because of severe growth deficiency, mild tetraparesis (force in upper limbs is up to 3 points, in lower limbs up to 3.5 points. She was able only to walk short distances within the room with a tendency to fall. In addition, a progressive kyphosis of the thoraco-lumbar spine was notable. In her first 2 years of life, her subsequent course of development has been described of being of moderate retardation in acquiring the skills of motor development because of her marked ligamentous hyperlaxity which was confused primarily with muscular dystrophy. The latter was the reason to keep the child under a series of investigations at the department of neurology, but muscular dystrophy could not be confirmed. Clinical examination showed a severe growth deficiency (minus 4 standard deviations = −4SD) and the head appeared large in comparison to the rest of her body (occipito-frontal circumference was plus 2SD). Craniofacially she manifested coarse facial features. The apparent skeletal abnormalities were severe ligamentous hyperlaxity, pectus excavatum and kyphosis of the thoraco-lumbar area. Interestingly, severe kyphosis with 48° at the level Th11 - L2 was evident. Radiological skeletal survey showed dysostosis multiplex. Full dynamic cervical spine radiographs showed atlanto-axial subluxation in connection with hypoplasia of the odontoid process. The clinical and the radiographic phenotypic characterizations were compatible with the Morquio syndrome (MPS type IV). Enzymatic tests revealed deficiency of the enzyme GALNS, which is essential for the degradation of keratan-sulfate and chondroitin-6-sulfate, confirmed by 2 mutations in the *GALNS*-gene confirming Morquio type A (MPS IV A). 3DCT-scan and magnetic resonance tomography-imaging (MRI) showed significant stenosis of the spinal canal up to 0.39 cm at the cervical spine level C1–C2, hypoplasia of the dens, presence of vertebrogenic myelopathy, and mechanical and neurological instability at the cranio-vertebral junction. Laminectomy of C1 was done at the Stage II of surgical treatment (occipito-spondylodesis). Correction and posterior instrumentation of the cervical spine at C0–C5 level in conjunction with posterior spinal fusion with costal autograft was performed. Few months after decompression at C1 level, occipitospondylodesis with fixation of the upper 2 or 3 cervical vertebrae to the occipital bone was performed (Fig. 6). A follow-up for a period of 6 months following the decompression at the level of C1, a remarkable improvement in her neurological status could be noticed. The above-mentioned procedures were followed by correction of the thoraco-lumbar kyphosis via posterior spinal fusion at the level of T6–L4 by means of “growing rods” system (Fig. 7). The girl began to walk independently and the strength of her upper limbs reached up to 4 points, which signify dramatic response to our surgical interventions. To improve her locomotor activity, correction of bilateral genu valgum was done by means of 8 plate fixation.

## 3 Discussion

Congenital or developmental cervical kyphosis might be seen in several syndromic entities, such as Larsen syndrome, diastrophic dysplasia, campomelic dysplasia, and in chondrodysplasia punctata. Congenital cervical instability and kyphosis is the consequence of the vertebral body hypoplasia associated with functional disconnection of the posterior elements of the spine related to underdeveloped pedicles.^[13]^

Partial or total vertebral body absence causing congenital kyphosis was first described by Rokitansky in 1844.^[14]^ Van Schrick in 1932 described the deformity as maldevelopment of the vertebral body centrum.^[15]^ He divided them into 2 basic groups, with either failure of segmentation of the vertebral body with adjacent fusion of anterior portions of the vertebral bodies or absence of vertebral body development. Winter and Moe^[16]^ described 130 cases of congenital kyphosis and proposed a new classification with 3 basic types. Type 1 means the absence of vertebral bodies, whereas type 2 is a failure of segmentation with accompanying kyphosis developing more gradually. Type 3 is a combination of both, type 1 and type 2, the kyphosis in the vast majority of these patients was in the thoraco-lumbar region.

The term “campomelic” refers to bowing of the long bones, primarily the tibiae and femora. Although campomelia is one of the most common clinical features of this disorder and the feature that gives it its name, cases without campomelia (“acampomelic” campomelic dysplasia) have been described. In those, neither lower limb bowing nor talipes equinovarus typical of campomelic dysplasia can be observed.^[1,17]^ Patients with CD are mostly manifesting severe cervcial kyphosis associated with cervical instability, although variable severity can be seen. But all patients develop a severe progressive thoracic kyphoscoliosis. Late ossification of the mid-thoracic pedicles is a clear and early diagnostic finding and vertebral body hypoplasia is the cause of deformity. Progression of the spine deformity adversely leads to pulmonary compromise and if untreated, mortality might be the outcome. ^[18]^ Coscia et al described significant spinal findings in 8 patients with campomelic dysplasia.^[19]^

The Larsen syndrome is a rare inherited defect of connective tissue that is transmitted in both an autosomal dominant and recessive pattern. First described by Larsen, its cardinal findings consist of multiple congenital joint dislocations, usually of the hips, knees, and elbows.^[5]^ Cervical kyphosis is the most serious of the wide variety of spine anomalies reported in Larsen syndrome. The incidence of cervical kyphosis is ∼12% in patients suffering from the Larsen syndrome. Progressive instability, myelopathy, and sudden death can occur. Therefore, the management of cervical kyphosis is the first priority to be applied in patients with the Larsen syndrome. MRI imaging is the modality of choice to determine the urgency of stabilization. Fusion is difficult in a child younger than 1 year, and bracing may be used until surgery is planned after age of 18 months.^[20]^ Posterior cervical fusion in infants younger than 1 year is associated with a definite risk of pseudoarthrosis and failure. The latter form of surgical intervention is preferable for patients with anteroposterior dissociation and absent pedicles, resulting in complete separation of the laminae and vertebral bodies at multiple levels.^[20]^

Johnston et al^[21]^ suggested that delaying posterior cervical fusion until about 18 months of age may result in higher rate of fusion and subsequent correction of the deformity by continued anterior growth in the presence of a solid posterior tether. Madera et al^[22]^ treated cervical instability with synchronous anterior decompression and fixation, posterior fusion and fixation, and halo placement. At 1-year follow-up, the child had improved cervical alignment with no associated neurologic deficits.

In the Morquio syndrome, hypoplasia of the odontoid process combined with ligament hyperlaxity and joint instablity leads to instability at the level of the first 2 cervical vertebrae, with a high risk of spinal cord compression. Spinal involvement in MPS IVA occurs at 2 distinct sites. Cervical spinal involvement, particularly instability and compression at the C1–2 level, is almost a unique finding and predisposes patients to severe neurological deficits, paralysis and sudden death. Spinal abnormalities are usually of progressive nature, but nevertheless, neurological deficits can occur suddenly.^[11]^ A combination of cervical spine instability and glycosaminoglycan deposition in the extradural soft tissues leads to upper cervical stenosis and consequently myelopathy.

Decompression and fusion may be preferred in an asymptomatic patient if the space available for the spinal cord is less than 14 mm, the C1–2 instability is more than 8 mm, or lateral cervical flexion-extension radiographs and dynamic MRI showed spinal cord impingements. Atlantoaxial instability in MPS IV A is treated by posterior fusion.^[23]^ Postoperative immobilization in a halo vest is necessary. If untreated, myelopathic changes progress rapidly and traumatic quadriparesis and death due to respiratory insufficiency have been described.^[24]^

## 4 Conclusion

In our current paper, we detailed the major imaging phenotype of 3 devastating syndromic entities and we described the applied tools to establish diagnosis and in some to control and lessens the outcome of the vicious spinal deformities.

The usage of CT scan and MRI imaging were modalities of choice for the localization and assessment of the cervical spine pathology.

Our patients manifested incomplete ossification of the cartilaginous vertebral bodies, resulting in mal-development of the spine segments. The incomplete ossification of the atlas, odontoid hypoplasia, and the accompanying laxity of the transverse and alar ligaments (particularly in MPS IVA), leads to atlanto-axial instability.

Syndromic recognition depends more often upon appropriate knowledge in clinical and radiographic phenotypic characterization.

Clinical skill and experience is the base line tool of management than upon technically complex laboratory investigations. Knowledge and understanding of the natural history of each syndromic entity is a requisite for proper management.
